# Protocol for a multi-phase, mixed methods study to develop and evaluate culturally adapted CBT to improve community mental health services for Canadians of south Asian origin

**DOI:** 10.1186/s13063-021-05547-4

**Published:** 2021-09-06

**Authors:** Farooq Naeem, Andrew Tuck, Baldev Mutta, Puneet Dhillon, Gary Thandi, Azaad Kassam, Nimo Farah, Aamna Ashraf, M. Ishrat Husain, M. Omair Husain, Helen-Maria Vasiliadis, Marcos Sanches, Tariq Munshi, Maureen Abbott, Nicholas Watters, Sean A. Kidd, Muhammad Ayub, Kwame McKenzie

**Affiliations:** 1 Department of Psychiatry, University of Toronto, Toronto, Canada; 2 Centre for Addiction and Mental Health, 33 Ursula Franklin St., Toronto, Ontario M5S 2S1 Canada; 3 Punjabi Community Health Services, Toronto, Canada; 4 Moving Forward Family Services, Vancouver, Canada; 5 University of Ottawa, Ottawa, Canada; 6 Somerset West Community Health Centre, Ottawa, Canada; 7 Department of Community Health Sciences, Faculty of Medicine and Health Science, Université de Sherbrooke, Research Center Charles-Le Moyne – Saguenay–Lac-Saint-Jean sur les innovations en santé, Montreal, Canada; 8 St Michael’s Hospital, Toronto, Canada; 9 Mental Health Commission of Canada, Ottawa, Canada; 10 Queen’s University, Kingston, Canada; 11 Wellesley Institute, Toronto, Canada

**Keywords:** South Asian, Culturally adapted cognitive Behavioural therapy, Canada, Depression, Anxiety

## Abstract

**Background:**

Canadians of South Asian (SA) origin comprise the largest racialized group in Canada, representing 25.6% of what Statistics Canada terms “visible minority populations”. South Asian Canadians are disproportionately impacted by the social determinants of health, and this can result in high rates of mood and anxiety disorders. These factors can negatively impact mental health and decrease access to care, thereby increasing mental health inequities. Cognitive Behavioural Therapy (CBT) in its current form is not suitable for persons from the non-western cultural backgrounds. Culturally adapted Cognitive Behavioural Therapy (CaCBT) is an evidence-based practice. CaCBT is more effective than standard CBT and can reduce dropouts from therapy compared with standard CBT. Thus, CaCBT can increase access to mental health services and improve outcomes for immigrant, refugee and ethno-cultural and racialized populations. Adapting CBT for growing SA populations in Canada will ensure equitable access to effective and culturally appropriate interventions.

**Methods:**

The primary aim of the study is to develop and evaluate CaCBT for Canadian South Asian persons with depression and anxiety and to gather data from stakeholders to develop guidelines to culturally adapt CBT. This mixed methods study will use three phases: (1) cultural adaptation of CBT, (2) pilot feasibility of CaCBT and (3) implementation and evaluation of CaCBT. Phase 1 will use purposive sampling to recruit individuals from four different groups: (1) SA patients with depression and anxiety, (b) caregivers and family members of individuals affected by anxiety and depression, (c) mental health professionals and (d) SA community opinion leaders. Semi-structured interviews will be conducted virtually and analysis of interviews will be informed by an ethnographic approach. Phase 2 will pilot test the newly developed CaCBT for feasibility, acceptability and effectiveness via quantitative methodology and a randomized controlled trial, including an economic analysis. Phase 3 will recruit therapists to train and evaluate them in the new CaCBT.

**Discussion:**

The outcome of this trial will benefit health services in Canada, in terms of helping to reduce the burden of depression and anxiety and provide better care for South Asians. We expect the results to help guide the development of better services and tailor existing services to the needs of other vulnerable groups.

**Trial registration:**

ClinicalTrials.gov NCT04010890. Registered on July 8, 2019

## Background

Canadians of South Asian (SA) origin^1^ comprise the largest racialized group in Canada, representing 25.6% of what Statistics Canada terms “visible minority populations”, followed by East Asian and Black Canadians, respectively [1]. South Asian Canadians are disproportionately impacted by the social determinants of health, including unemployment, low income, language barriers, low education, low literacy and migration stress. This can result in high rates of mood and anxiety disorders, with SA individuals who immigrate to Canada at age 17 or younger at a significantly higher risk compared to immigrants who came to Canada when they were 18 or older [2, 3].

These factors can negatively impact mental health and decrease access to care, thereby increasing mental health inequities [4]. Compared to other ethno-cultural groups, SA Canadians with a major depressive episode reported the highest proportion of unmet mental health care needs (48%) and the highest percentage of perceived barriers to the availability of mental health care (33%) [5]. Canadians who had a major depressive episode and identified as SA were 85% less likely to seek treatment than Canadians who had experienced the same illness but identified as white [4]. The lower use of mental health services by SA Canadians highlights the inequities in access to appropriate care for these populations [4–7].

The Mental Health Commission of Canada [MHCC] [8] recommends improving Canadian mental health care to serve diverse populations with equitable, timely access to appropriate, effective and evidence-based treatments that attend to unique sociocultural needs.

The MHCC Case for Diversity [4] report further highlights the necessity for culturally and linguistically relevant services particularly for immigrant, refugee and ethno-cultural and racialized populations, such as SA Canadians. In light of the new $5 billion targeted federal transfer to “improve access to mental health and addiction services and to structured psychotherapy” [9], there is an opportunity to complement efforts to expand the access that adequately address the mental health needs of diverse Canadian populations.

Cognitive Behavioural Therapy (CBT) in its current form is not suitable for persons from the non-western cultural background [10–13]. Culturally adapted Cognitive Behavioural Therapy (CaCBT) is an evidence-based practice [10, 14–18]. CaCBT is more effective than standard CBT and can reduce dropouts from therapy compared with standard CBT [19, 20]. Thus, CaCBT can increase access to mental health services and improve outcomes for immigrant, refugee and ethno-cultural and racialized populations [21–23]. Adapting CBT for growing SA populations in Canada will ensure equitable access to effective and culturally appropriate interventions. Accordingly, this study proposes to develop and evaluate CaCBT for depression and anxiety among SA populations in Canada.

### Aims and objectives

The primary aim of the study is to develop and evaluate CaCBT for Canadian South Asian persons with depression and anxiety and to gather qualitative data from stakeholders to develop guidelines to culturally adapt CBT. The secondary objectives include (a) evaluate the feasibility and acceptability of CaCBT, (b) conduct an economic evaluation of CaCBT and (c) test whether training in culturally adapted CBT can improve therapists’ cultural competence.

## Methods

This mixed methods study will be conducted in three phases.

### Phase 1: cultural adaptation of CBT

#### Cultural adaptation of CBT for SA populations in Canada experiencing depression and anxiety using stakeholder consultations and qualitative methodology

##### Methods

Key stakeholders from various SA communities living in the Greater Toronto Area (GTA) (municipalities of Halton, Peel, York, Toronto and Durham), Vancouver and Ottawa will be engaged to inform the development of CaCBT guidelines for depression and anxiety based on previously developed qualitative methodology [24–28]. Semi-structured interviews [29, 30] will be guided by predetermined themes based on previous qualitative research.

##### Recruitment

A purposive sampling method will be used to recruit individuals from four different target groups: (a) SA patients with depression and anxiety (*n* = 60), (b) caregivers and family members of individuals affected by anxiety and depression (*n* = 60), (c) mental health professionals (*n* = 30) and (d) SA community opinion leaders (*n* = 30) through the partnering agencies and within the local communities. Snowball sampling will be used to facilitate recruitment to fulfil our total sample size for each target group [29]. We will recruit individuals who help to represent the diversity within the SA population in Canada, including first-, second- and third-generation SA Canadians. Furthermore, recruitment materials will be developed in various SA languages such as Hindi, Punjabi, Urdu, Bengali, Nepali, Pashto, Burmese, Tamil and Farsi to engage SA individuals whose first language is not English or French. Special attention will be given to recruitment methods to ensure gender parity in the sample population. For example, we aim to recruit female participants from local religious institutions, SA women’s centres and associations and through local South Asian female community leaders.

##### Data collection

Potential participants will be given information about the study and screened for eligibility. Eligible participants who provide informed consent will be invited to participate. Semi-structured interviews will last 60 min and be held virtually. Interviews will be audio recorded, fully transcribed and checked for accuracy. Participants will be informed of their right to withdraw from the study before, during and after data collection. Interviews will be conducted by research assistants who will receive regular supervision from the research team. Since sex/gender interactions can affect the type and quality of information disclosed in qualitative research settings, special attention will be paid to gender equality among our participants. All interviews will be conducted through the WebEx platform, following local privacy protocols.

##### Data analysis

The analysis will be informed by an ethnographic approach [31] using the principle of emergent design [32]. Collected data will be analysed for systematic content and themes. Researchers will immerse themselves in the data by carefully reading transcripts several times and identifying emerging themes and categories [33, 34]. Regular research team meetings will be held throughout data analysis, facilitating further exploration of participants’ responses, discussion of deviant cases and agreement on recurring themes. Identified themes will be converted into codes and will be organized into wider themes and categories (e.g. barriers to therapy). Nvivo 9 software will be used to facilitate the analytical process. Each research assistant involved in collecting data will begin analysis as interviews are being conducted. Triangulation of themes and concepts will be undertaken by comparing themes from different participant groups to test the validity of the data. Three research assistants will independently analyse randomly selected transcripts using the thematic framework and compare results to further test validity.

The analysis process will be completed once saturation has been reached. However, given limited resources and based on our previous experience, only a certain number of interviews will be conducted. Wider themes and categories will be developed based on the results from the qualitative data. An existing standard CBT manual developed by the principal applicant [35] will be culturally adapted by the research team using the qualitative results with important cultural aspects as identified in the data integrated throughout (e.g. centrality of religion, extended family structures etc.).

### Phase 2: pilot feasibility testing of CaCBT

#### Pilot test the newly developed CaCBT for feasibility, acceptability and effectiveness via quantitative methodology and a randomized controlled trial

##### Methods

The newly developed CaCBT will be pilot tested for feasibility, acceptability and effectiveness and compared to standard CBT at three sites in Canada (GTA, Ottawa and Vancouver). A randomized controlled assessor-blind clinical trial will compare CaCBT with standard CBT.

Participants who meet the inclusion criteria will be randomly allocated to one of two groups at each site: CaCBT (experimental group) or standard CBT (control group) in a 1:1 ratio. The experimental group will receive 8–12 weekly sessions of the newly developed CaCBT while the control group will receive 8–12 weekly sessions of standard CBT. Therapists providing both CaCBT and standard CBT will be trained to ensure the fidelity of the therapies being offered.

##### Trial status

The trial was registered with clinicaltrials.gov NCT04010890. Registered on July 8, 2019. Recruitment for the trial will begin in March 2021 and is expected to be completed by September 2021.

##### Trial intervention

CaCBT will be delivered to the experimental group using the newly developed manual. The intervention will be delivered over 8–12 sessions. The control group will receive standard CBT.

##### Timeline

**Fig. 1:**
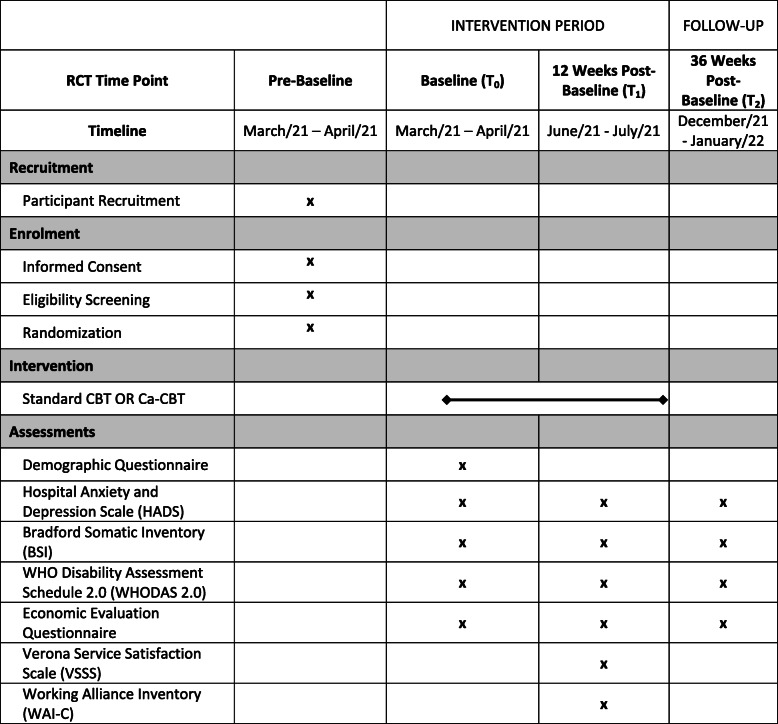
Participant enrollment and intervention schedule

The timeline is presented in Fig. 1.

##### Training and fidelity

Therapists will be employed at participating agencies and will be accredited professionals who have experience delivering CBT. Six part-time therapists will undergo 3 days of intensive training by FN for either CaCBT or standard CBT. The training will be provided separately to prevent cross-contamination between the two groups. Therapists will be assigned to provide either CaCBT to the experimental group or standard CBT to the control group. Therapists will be under group supervision by FN via Internet video-conferencing every week. Protocol adherence and treatment integrity will be assessed using the revised Cognitive Therapy Scale (CTS-R) [36].

##### Participants

Depressed or anxious SA participants will be recruited from participating hospitals or primary health care centres in the identified cities. Participants, between the ages of 18 and 64 who score 8 or more on the depression subscale or the anxiety subscale of the Hospital Anxiety and Depression Scale (HADS), will be invited to participate in the study. Participants, who use alcohol or drugs excessively, have significant cognitive impairment (e.g. intellectual disability or dementia) or have active psychosis, using DSM-5 criteria, will be excluded. Similarly, participants who have received CBT during the previous 12 months will also be excluded. If any participant requires care after the trial has completed, they will be directed to contact their therapist.

##### Sample size calculation

The sample size was calculated using a comparison between group change in the HADS Depression subscale scores. With a 5% significance level and 90% power, we determined that 48 subjects per group will be required for the trial, in order to detect a standardized effect size Cohen’s *d* = 0.68, which is equivalent to a difference in HADS score at the end of the trial of around 2.4 points, assuming a standard deviation of 3.5 found in previous studies from our lab that used the same outcome. To account for up to 30% attrition, we plan to recruit a total of 140 participants into the study.

##### Randomization procedure

A statistician blind to the recruitment process will provide the randomization schedule. The randomization will be conducted in blocks of size 2, 4, 6 or 8, with size of blocks defined randomly, and stratified by gender and by site, to ensure balance in treatment assignment and gender. Assignment will be concealed meaning that the person assigning subjects to arms will not be aware of the assignment until the moment it happens. This will be achieved by having individual assignments in sealed pre-ordered and numbered envelopes. Subjects who do not identify themselves as male or female will be randomized to one of these two genders using a parallel randomization schedule. A statistician will create the randomization schedule in R, using a seed so that the process is reproducible. The statistician will then prepare the sealed envelopes along with instructions for the randomization. Participants will also be blinded to allocation as they both will receive CBT.

##### Measurements

Assessments will be carried out at baseline after therapy has been completed (12 weeks from baseline) and at follow-up (36 weeks from baseline) by raters blinded to group allocation. The primary outcome, the effectiveness of the psychotherapy addressing levels of depression and anxiety, will be measured using the HADS Depression subscale [37]. HADS is a 14-item, self-assessment scale designed to measure anxiety and depression. The maximum score is 21 for depression and 21 for anxiety. A score of 8–10 suggests the presence of the borderline cases, while a score of 11–21 indicates probable cases. Secondary outcomes, including somatic symptoms, disability, feasibility, acceptability and satisfaction, will also be measured. The Bradford Somatic Inventory (BSI) enquires about a wide range of somatic symptoms during the previous month and has 45 items. Scores above 21 indicate depression [38]. Disability will be measured using the World Health Organization’s Disability Assessment Schedule 2.0 (WHODAS) [39]. This scale assesses disability due to physical and psychological problems and has been used extensively in various research settings. Feasibility will be measured through engagement, recruitment and retention of participants. Acceptability will be measured through feedback from participants and therapists. Participants will also be asked to report their satisfaction by responding to the Verona Service Satisfaction Scale [40]. The Working Alliance Inventory (WAI) [41] will be used to measure therapeutic alliance.

A questionnaire adapted from Beecham and Knapp’s [42] costing psychiatric interventions measurement protocol will be used to ascertain health and social service utilization relevant from the perspective of the health system and participant perspectives. The questionnaire filled out by participants at baseline will inquire on service utilization in the past 6 months. The questionnaires at 3 and 9 months post-treatment will inquire about service utilization in the past 3 and 6 months. Information on outpatient and emergency department visits, hospitalizations and medications, visits to community organizations and receipt of social services will be collected. Participant out-of-pocket costs for seeking health and social services will also be considered and include drug co-payments, direct payments to professionals not covered by public insurance plan, cost related to transportation and time spent by patients while consulting.

The CaCBT programme costs associated with the training of professionals and therapy sessions provided to participants in the CaCBT arm will be considered and will include professional salaries and benefits and institutional overhead and building opportunity costs.

The cost analysis in this study will be carried out from the health system and participant perspectives. Participant costs will be based on the measurement and costing of health resources according to published Canadian guidelines [43] and published methodology in evaluating mental health programmes and attributable costs associated with mental disorders in Canada [44–48].

##### Data collection

Potential participants will be consented by the research assistants. The study research assistants will collect the data, some of it will be collected through REDCap and all data will be shared by the hospital’s secure file transfer process.

##### Data monitoring

Data will be stored on a secure server at the hospital. The data is backed up regularly. The access to the server will be strictly password protected. We are working closely with hospital IT and following SOPs. A data dictionary will be created to technically define the databases, its tables and structure. Combined with annotated and coded case report forms, this will provide a curated set of data.

A Data and Safety Monitoring Team consisting of the applicants and research manager is established for oversight and monitoring of the conduct of the trial. This team meets twice a year to ensure the safety of participants and the validity and integrity of the data.

All data breaches will be reported to the hospital research ethics committee.

##### Statistical analyses

We will follow the CONSORT guidelines for randomized controlled trials [49]. The statistical analysis of the data will be conducted by a statistician blinded to the treatment group. The primary outcomes will be the total HADS score and secondary analysis will be conducted using the Anxiety and Depression subscales. Intention to treat will be used for our primary analysis, where subjects will be analysed in the groups they were randomized regardless of compliance of having dropped out. The mixed effect model, where subjects are the random effects, time (baseline, 12 weeks and 36 weeks) and group (CaCBT and Standard CBT) as well as the interaction between time and group will be entered as fixed factors, with gender and baseline HADS as covariates. The significance of the interaction between group and time tests for difference in HADS score at 3 or 6 months and will be used to declare the intervention effective. Since recruitment will be conducted in 3 sites, these sites will also enter the model as random effects. We will also control for therapists variation by coding them as a variable in the dataset, then adding them to the model as a random effect. Mixed effect models estimated through maximum likelihood are able to account for dropouts under the assumption they are missing at random (MAR) [50, 51]. Comparison between dropouts and completers will be conducted at baseline to look at evidences of differences between them, and if significant differences are found, they will be accounted for by adding the variable as a control in the model. A significance level of 0.05 will be used to declare the CaCBT performs better than standard CBT. A diagnostic analysis will be conducted using Cook’s distance to detect possible influential data points, and if any is found, results without these subjects will be also presented if they lead to meaningfully different conclusions. Residuals will be checked for heterogeneity of variance that, if found, will be addressed by the use of robust “sandwich” type standard errors. We may also conduct a sensitivity analysis using completers only with ANCOVA and inverse propensity weighting, where weights are the estimate probability of dropping out estimated by a logistic regression model having baseline characteristics as predictors of the subjects. Such ANCOVA has the HADS score at the end of the trial as the outcome and HADS at baseline as the covariate, while also controlling for gender and sites. This approach is suggested in [52]. Finally, a similar ANCOVA without weights will be conducted using last observation carried forward (LOCF) and all subjects. If more than 10% of the subjects are found to be non-compliant, a per-protocol analysis will also be conducted where non-compliant subjects are removed. All these extra analyses will provide information on the robustness of the primary analysis through the mixed effect model.

For the economic evaluation, an incremental cost-effectiveness analysis will be carried out to estimate the cost-effectiveness (C/E) ratio associated with the CaCBT programme according to published guidelines [53, 54]. The costs incurred during the study period (3 and 9 months) will be accounted for in both experimental arms. Effectiveness will be considered based on a 25% decrease in HADS and improvement (yes/no) in WHODAS scores between baseline and the 3-month and 9-month observation periods. The C/E ratio will be calculated for the 3-month and 9-month observation periods as follows: [average costs (CaCBT group) – average costs (control group)]/[# of improvements in CaCBT group − # of improvements in control group]. This will report the additional average costs associated with one improved case associated with the programme. Treatment groups will be compared with respect to baseline characteristics (age, sex) as well as scores on the HADS, WHODAS and health service utilization in the previous 6 months such as the number of outpatient and emergency department visits and hospitalizations and follow-up and adherence to programme. This will elucidate whether these factors will need to be considered in the analyses and interpretation of C/E results. Sensitivity analyses will also be carried out to test the robustness of the results.

### Phase 3: implementation and evaluation of CaCBT

#### Trained therapists working with SA populations to use CaCBT with their clients. Evaluate therapist competence in using CaCBT as well as client satisfaction with the newly developed therapy

##### Methods

The newly developed CaCBT guidelines will be evaluated for enhancing cultural competence among therapists already delivering CBT.

##### Participants

Twenty to thirty therapists not involved in the previous phases will be recruited from participating hospital and community organizations and will attend one full-day training course in CaCBT. We will use a convenient sampling for this recruitment.

##### Measurements

Participants’ knowledge and attitudes will be assessed using pre- and post-training scores on a questionnaire derived from an existing competence in culturally adapted therapy framework [22]. A visual analogue scale will be used to measure their satisfaction and perceived competence before and after training. We will also gather post-training feedback from participating therapists to further enhance our understanding.

##### Analyses

SPSSv25 will be used to measure the change in pre- and post-training scores in therapist competence and their satisfaction using the *t*-test and chi-square test.

## Discussion

Research shows high rates of depression and anxiety among SA Canadians. This group also has lower rates of access to mental health care and has poorer outcomes. There are a number of psychosocial treatments available for depression and anxiety, but to the best of our knowledge, there has been no trial of any evidence-based intervention in this hard-to-reach group in Canada. As such, this is the first trial of culturally adapted CBT for South Asians in North America and Europe. This paper describes the development and testing of culturally adapted CBT. As far as we are aware, this is the first fully powered trial to compare culturally adapted CBT with standard CBT and to conduct an economic evaluation.

The impact of this intervention on service users will be to improve their mental health literacy and improve their self management capabilities through CBT. This may lead to improvement in their well-being and improve their satisfaction with health services as well as improvements in relationships, quality of life and functioning. We are undertaking a detailed process of culturally tailoring this intervention to the specific needs of our target population. This will not only help in improved efficacy but will possibly lead to fewer dropouts and higher satisfaction. The results of this study will be shared widely. Using an integrated dissemination plan, the results of the various stages will be shared through a variety of means including a launch event, webinars, manuscripts and policy reports.

The outcome of this trial will benefit health services in Canada, in terms of helping to reduce the burden of depression and anxiety and provide better care for South Asians. We expect the results to help guide the development of better services and tailor existing services to the needs of other hard-to-reach and vulnerable groups and for other cultural populations.

## Abbreviations

BSI: Bradford Somatic Inventory
CaCBT: Culturally adapted Cognitive Behavioural Therapy
CBT: Cognitive Behavioural Therapy
C/E: Cost-effectiveness
GTA: Greater Toronto Area
HADS: Hospital Anxiety and Depression Scale
LOCF: Last observation carried
MAR: Missing at random
MHCC: Mental Health Commission of Canada
SA: South Asian
WAI: Working Alliance Inventory
WHODAS: World Health Organization Disability Assessment Schedule

## Footnotes

1 The population for this project does not only include Canadian citizens, but also immigrants, refugees and newcomers who do not have Canadian citizenship.
